# Optical Recording of Action Potentials in Human Induced Pluripotent Stem Cell-Derived Cardiac Single Cells and Monolayers Generated from Long QT Syndrome Type 1 Patients

**DOI:** 10.1155/2019/7532657

**Published:** 2019-03-06

**Authors:** Tadashi Takaki, Azusa Inagaki, Kazuhisa Chonabayashi, Keiji Inoue, Kenji Miki, Seiko Ohno, Takeru Makiyama, Minoru Horie, Yoshinori Yoshida

**Affiliations:** ^1^Department of Cell Growth and Differentiation, Center for iPS Cell Research and Application, Kyoto University, Sakyo-ku, Kyoto 606-8507, Japan; ^2^Department of Cardiology, Japanese Red Cross Kyoto Daini Hospital, Kamigyo-ku, Kyoto 602-8026, Japan; ^3^Department of Bioscience and Genetics, National Cerebral and Cardiovascular Center Research Institute, Suita, Osaka 565-8565, Japan; ^4^Department of Cardiovascular Medicine, Kyoto University Graduate School of Medicine, Sakyo-ku, Kyoto 606-8501, Japan; ^5^Center for Epidemiologic Research in Asia, Shiga University of Medical Science, Seta-Tsukinowa-cho, Otsu 520-2192, Japan

## Abstract

Induced pluripotent stem cells (iPSCs) from type 1 long QT (LQT1) patients can differentiate into cardiomyocytes (CMs) including ventricular cells to recapitulate the disease phenotype. Although optical recordings using membrane potential dyes to monitor action potentials (APs) were reported, no study has investigated the disease phenotypes of cardiac channelopathy in association with the cardiac subtype at the single-cell level. We induced iPSC-CMs from three control and three LQT1 patients. Single-cell analysis using a fast-responding dye confirmed that ventricular cells were the dominant subtype (control-iPSC-CMs: 98%, 88%, 91%; LQT1-iPSC-CMs: 95%, 79%, 92%). In addition, LQT1-iPSC-ventricular cells displayed an increased frequency of early afterdepolarizations (*p*value = 0.031). Cardiomyocyte monolayers constituted mostly of ventricular cells derived from LQT1-iPSCs showed prolonged AP duration (APD) (*p*value = 0.000096). High-throughput assays using cardiomyocyte monolayers in 96-well plates demonstrated that I_Kr_ inhibitors prolonged APDs in both control- and LQT1-iPSC-CM monolayers. We confirmed that the optical recordings of APs in single cells and monolayers derived from control- and LQT1-iPSC-CMs can be used to assess arrhythmogenicity, supporting the feasibility of membrane potential dye-based high-throughput screening to study ventricular arrhythmias caused by genetic channelopathy or cardiotoxic drugs.

## 1. Introduction

Type 1 long QT syndrome (LQT1), a frequent type of congenital LQT syndrome (cLQTS) [1], is caused by a reduction of slow delayed rectifier K^+^ current (I_Ks_) and is associated with a loss-of-function mutation in the *KCNQ1* gene [2]. *KCNQ1* A341V is known as one of the most frequent and severe *KCNQ1* mutations [3]. Its coexpression with wild-type *KCNE1*, a beta subunit of the I_Ks_ channel, causes a pathological reduction of I_Ks_ [4], suggesting the importance of building physiological conditions to recapitulate the pathology in vitro. Human induced pluripotent stem cell-derived cardiomyocytes (hiPSC-CMs) were shown to remodel ion channels that regulate the electrophysiological activity of the human heart and to successfully recapitulate the LQT1 phenotype [5–8].

Previous papers related to cLQTS patient-specific hiPSC-CMs verified the disease phenotype by using either the manual patch clamp method alone [5, 7] or in combination with the microelectrode array (MEA) system [6, 8]. However, the field potential duration (FPD), which reflects QT on electrocardiograms (ECG), of ventricular hiPSC-CM monolayers is longer than that of atrial iPSC-CMs when using the MEA system [9], suggesting a high proportion of ventricular hiPSC-CMs is required to analyze ventricular arrhythmic disease. Patch clamp analysis is standard for classifying the cardiac subtype, but it requires much time and expertise [10]. Automated patch clamp techniques may resolve these drawbacks, but they cause cells to produce spontaneous action potentials (APs) that are erratic due to enzymatic treatment immediately before the analysis [11].

FluoVolt (FV) is a new membrane potential VF2.4.CI dye [12, 13]. It modulates photo-induced electron transfer (PeT) from an electron donor to a fluorophore through a synthetic molecular wire [12, 14] and responds on a microsecond timescale, which makes it faster than genetically encoded voltage indicators [15, 16]. Moreover, it has a higher fluorescence ratio (about 20% ΔF/F per 100 mV greater) and lower phototoxicity than electrochromic dyes such as di-4-ANEPPS [12, 17], enabling classification of the subtype of single cardiomyocytes [18].

FV and VF2.1.Cl have been used to analyze APs from iCell^®^ cardiomyocyte monolayers or type 3 LQTS-iPSC-CM monolayers and also drug effects on multiwell plate readers [19, 20]. However, the proportion of ventricular-type cells in those monolayers was not examined. Since arrhythmogenic change occurs in ventricular cells in LQTS, it is crucial to investigate the electrophysiological properties of this specific subtype.

In the present study, we propose a new method that incorporates FV and combines optical measurements of the membrane potential of single cardiomyocytes and in cardiac monolayers to identify the subtype and associate the subtype with the cardiomyocyte properties. We show the effectiveness of this method for modeling LQT1 using patient hiPSC-CMs.

## 2. Materials and Methods

### 2.1. Generation of Human iPS Cells from Three Type 1 LQTS Patients

This study was approved by the Ethics Committees of the Graduate School of Medicine Kyoto University and the Kyoto University Hospital. Written informed consent was obtained from the patients in accordance with the Declaration of Helsinki. LQT1-iPSCs were generated from three patients using episomal vectors as described previously [21]. Three iPSC lines derived from healthy donors (201B7 [22], 409B2 [21], and 692D2 [23]) were used as control iPSCs. Human iPSCs were maintained on STO feeder layers cultured with primate ES cell medium (ReproCell, Yokohama, Kanagawa, Japan), as previously described [22].

### 2.2. Cardiac Differentiation and Fluorescence-Activated Cell Sorting

Human iPSCs were differentiated by forming embryoid bodies (EBs), as previously described [24, 25] (see Supplementary Materials for further details). On day 29, EBs were dissociated and dispersed onto a fibronectin (Sigma-Aldrich, St Louis, MO, USA)-coated 6 cm dish. On the following day, seeded cells were collected by Accumax (Innovative Cell Technologies, San Diego, CA, USA) for 10 minutes and subjected to fluorescence-activated cell sorting (FACS) (Aria II, BD Biosciences, San Jose, CA, USA). To purify cardiomyocytes, SIRPa-positive and lineage (CD31, CD49a, CD140b, CD90, or TRA-1-60)-negative cells were sorted [26] and cryopreserved with STEM-CELLBANKER (Nippon Zenyaku Kogyo, Koriyama, Japan) at −80°C. A few weeks later, the cryotubes were transferred to a liquid nitrogen storage tank.

### 2.3. Quantitative Polymerase Chain Reaction (qPCR)

Total RNA was extracted after FACS using QIAzol Lysis Reagent (QIAGEN, Hilden, Germany). qRT-PCR was performed with TaqMan gene expression assays (Thermo Fisher Scientific, Waltham, MA, USA). For further details, see Supplementary Materials.

### 2.4. Thawing Frozen iPSC-CMs

For perforated patch clamp recording, the cryotubes were thawed in a 37°C water bath, centrifuged at 300 g for 5 min, and seeded on fibronectin-coated No. 2 cover glass (Matsunami, Osaka, Japan) filled with StemPro 34 SFM (Thermo Fisher Scientific) containing buffer and 10 ng/ml VEGF. Five to ten days after seeding, the cells were subjected to patch clamp experiments.

### 2.5. Current Clamp Recording

Perforated whole-cell patch was performed with current clamp mode using amphotericin B (Sigma-Aldrich), an Axopatch 200B amplifier (Molecular Devices, Sunnyvale, CA, USA), and pCLAMP software (Molecular Devices). Maximum diastolic potential (MDP), AP amplitude (APA), and action potential duration (APD) at 90% repolarization (APD_90_) were calculated from the average AP of 10 consecutive and stable waves in 1 Hz pacing mode with pCLAMP software. For detailed protocols, see Supplementary Materials.

### 2.6. Voltage Clamp Recording

I_Ks_ currents were recorded from single cardiomyocytes with the ruptured whole-cell patch clamp technique. I_Ks_ was calculated by subtracting the currents after perfusion with extracellular solution containing 30 *μ*M (-)-[3R,4S]-chromanol 293B (Tocris Bioscience, Bristol, UK) from the currents before perfusion. I_Ks_ currents were elicited by depolarizing steps from a holding potential of −40 mV to −20, 0, 20, and 40 mV for 4 s. This was followed by a 2 s repolarization phase. For detailed protocols, see Supplementary Materials.

### 2.7. Seeding High-Density iPSC-CM Monolayer

2 *μ*l of 50 *μ*g/ml fibronectin was placed on a 35 mm diameter glass bottom dish (Matsunami), or 5 *μ*l was placed on a 96-well plate with clear flat bottom wells (Corning, Corning, NY, USA). One hour later, fibronectin was aspirated, and a 2 *μ*l bead of thawed 4 × 10^4^ iPSC-CM suspension was placed on the 35 mm dish or a 5 *μ*l bead of thawed 5 × 10^4^ iPSC-CM suspension was placed on the 96-well plate. After another one-hour incubation, the appropriate volume of media was added gently. The dish or plate was incubated at 37°C, 5% CO_2_. The composition of the media was the same on days 7 to 29. Medium was exchanged every 2 to 3 days.

### 2.8. Loading of FV

When loading FluoVolt (FV; Thermo Fisher Scientific), the medium was exchanged with modified Tyrode's solution and FV (0.1% volume). Pluronic surfactants were not used in this study. Twenty minutes after loading, the medium was refreshed with modified Tyrode's solution and used in each assay.

### 2.9. Optical Recording of APs from Single Cells or Cardiomyocyte Monolayers

Cells were imaged in a 35 mm diameter glass bottom dish at 37°C and modified Tyrode's solution identical to that used for patch clamp recordings. Subarray images were recorded every 8 ms in single cells or every 4 ms in monolayer using AquaCosmos software (Hamamatsu Photonics). The regions of interest (ROIs) in the monolayers were defined as whole pixels of 512 × 32 at 1 Hz pacing. Graphing and calculation of APD_90_ were performed with OriginPro 2016 (OriginLab, Northampton, MA, USA). For further details, see Supplementary Materials.

### 2.10. Optical Recording of APs from Cardiomyocyte Monolayers on a High-Throughput Plate Reader

The FDSS/*μ*Cell imaging platform (Hamamatsu Photonics) was used. The LED current was set to 300 mA. An output excitation wavelength was used with standard settings of 492 BP 20 nm and emission filter 540AF40 (Omega Optical, Brattleboro, VT, USA). Binning was set to 4 × 4, and the measurement interval was every 4 ms. Cardiomyocyte monolayers 7–10 days after seeding on each well were stimulated at 1 Hz with 1 ms depolarizing pulses at 10 V. Analysis of APD_90_ was performed with FDSS Waveform Analysis software for cardiomyocytes (U8524-12; Hamamatsu Photonics).

### 2.11. Movie

Blinking monolayers were recorded with AquaCosmos software at 30 fps.

### 2.12. Statistical Analysis

All statistical analyses were verified with an unpaired *t*-test using Excel 2016 (Microsoft, Redmond, WA, USA). Values considered statistically significant are denoted as ^∗^ *p* < 0.05 and ^∗∗^ *p* < 0.005 in the figures.

## 3. Results

### 3.1. Establishment of Three LQT1 Patient-Derived iPS Cell Lines

**Figure 1:**
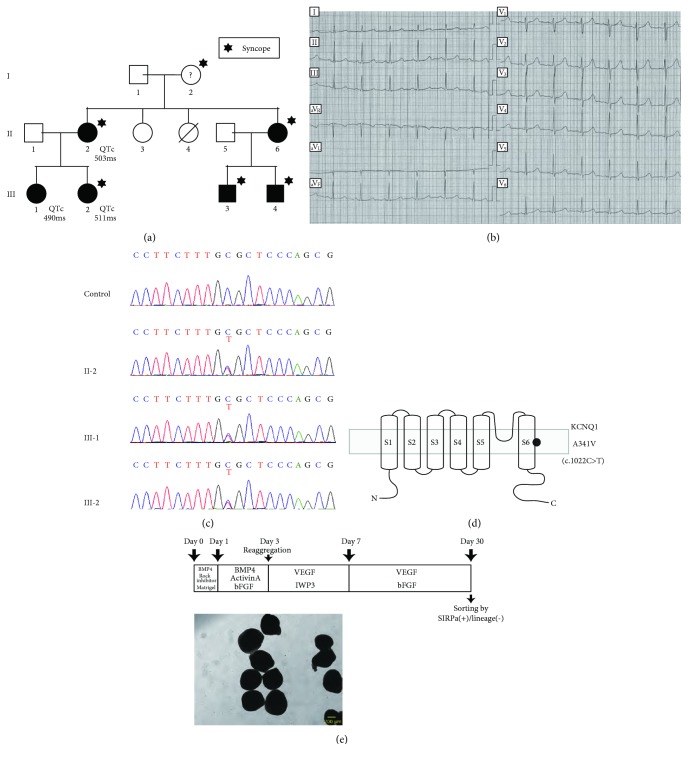
Type1 long QT syndrome family background and cardiac differentiation from human iPSCs. (a) Family pedigree. The squares indicate males and the circles indicate females. Closed symbols mark patients confirmed by their DNA sequences. Hexagrams mark members who have a syncope history. The QTc values of three patients before taking a beta-blocker are stated. (b) ECG of II-2 in Figure 1(a) before the patient started taking a beta-blocker. (c) Sanger sequencing of the three patients and one control. (d) Schematic figure of KCNQ1 protein. The black circle indicates the mutation site within the transmembrane region. The lower side locates intracellular. (e) Outline of the cardiac differentiation. Lower, representative shapes of beating EBs. Scale bar, 200 *μ*m.

We selected three LQT1 patients as donors for the iPSC derivation. One of the donors was a 50-year-old woman (II-2 in Figure 1(a)) who experienced presyncope several times when she was in junior high school and underwent recurrent syncope in her thirties. She showed prominent QT prolongation in resting ECG (Figure 1(b)) and exercise ECG. The other donors were her two daughters whose QT intervals were prolonged according to school medical examinations. Genetic testing diagnosed the mother and two daughters as type 1 long QT syndrome with *KCNQ1* A341V mutation (c.1022C>T) (Figure 1(c)). The mutation is located at the transmembrane region in segment 6 near the pore of the I_Ks_ channel (Figure 1(d)) and is reported as one of the severest types of LQT1 [3]. Medical therapy (beta-blockade) and lifestyle measures were sufficient to prevent recurrent events in the three patients. Five of the six family members positive for *KCNQ1* mutation experienced syncope, and the sixth (III-1 in Figure 1(a)) did not. All carriers showed QTc prolongation on ECG.

iPSCs were generated from the peripheral blood of the three patients using a nonviral method [21] and were differentiated into cardiomyocytes via EB formation [24, 25] (Figure 1(e)). There was no significant difference in the expression of genes that affect APD between control- and LQT1-iPSC-CMs (Supplementary Figure 1).

### 3.2. Patch Clamp Analysis

**Figure 2:**
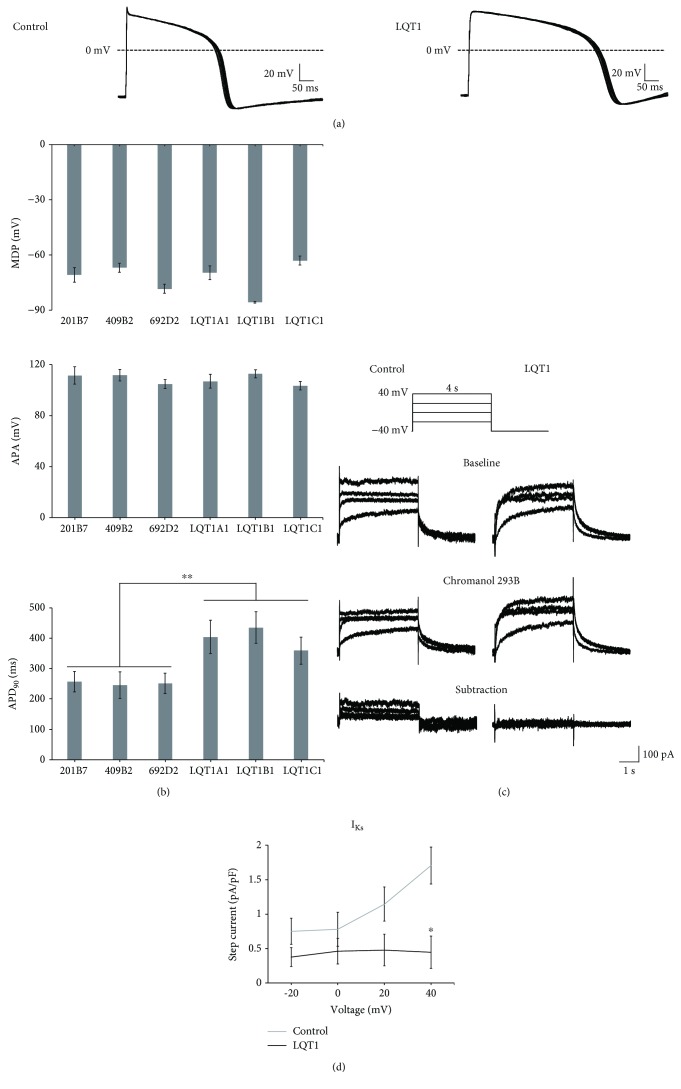
Patch clamp analysis of cardiomyocytes from control- and LQT-iPSC lines. (a) Representative APs of 1 Hz paced a control-iPSC-CM and an LQT-iPSC-CM from II-2 in Figure 1(a). Ten consecutive waves are shown. (b) MDP, APA, and APD_90_ from cardiomyocytes derived from the six lines: 201B7 (*n* = 6), 409B2 (*n* = 5), 692D2 (*n* = 6), LQT1A1 (*n* = 5), LQT1B1 (*n* = 5), and LQT1C1 (*n* = 7). Data are represented as means ± SEM; ^∗∗^ *p* < 0.005. (c) Representative current traces from control- and LQT1-iPSC-CMs. Upper, the protocol in current clamp recording. Middle, representative traces before and after perfusion with 3R4S-chromanol 293B (30 *μ*mol/l). Lower, 3R4S-chromanol 293B-subtraction. (d) I-V plots of I_Ks_ at the end of the depolarizing step. 692D2 (control) (*n* = 3), LQT1B1 (*n* = 3); ^∗^ *p* < 0.05.

Ventricular-type cardiomyocytes derived from the three control- and three LQT1-iPSC lines were subject to current clamp recordings (Figure 2(a)). Although there was no significant difference in MDP or APA, there was a significant difference in APD_90_ during pacing at 1 Hz between control- and LQT1-iPSC-CMs (Figure 2(b)) (*p* value, 0.0026). In addition, voltage clamp recordings revealed much smaller chromanol 293B-sensitive I_Ks_ currents from LQT1-iPSC-CMs than controls (Figures 2(c) and 2(d)).

### 3.3. Action Potentials Recorded by FV Dye in Single hiPSC-CMs

**Figure 3:**
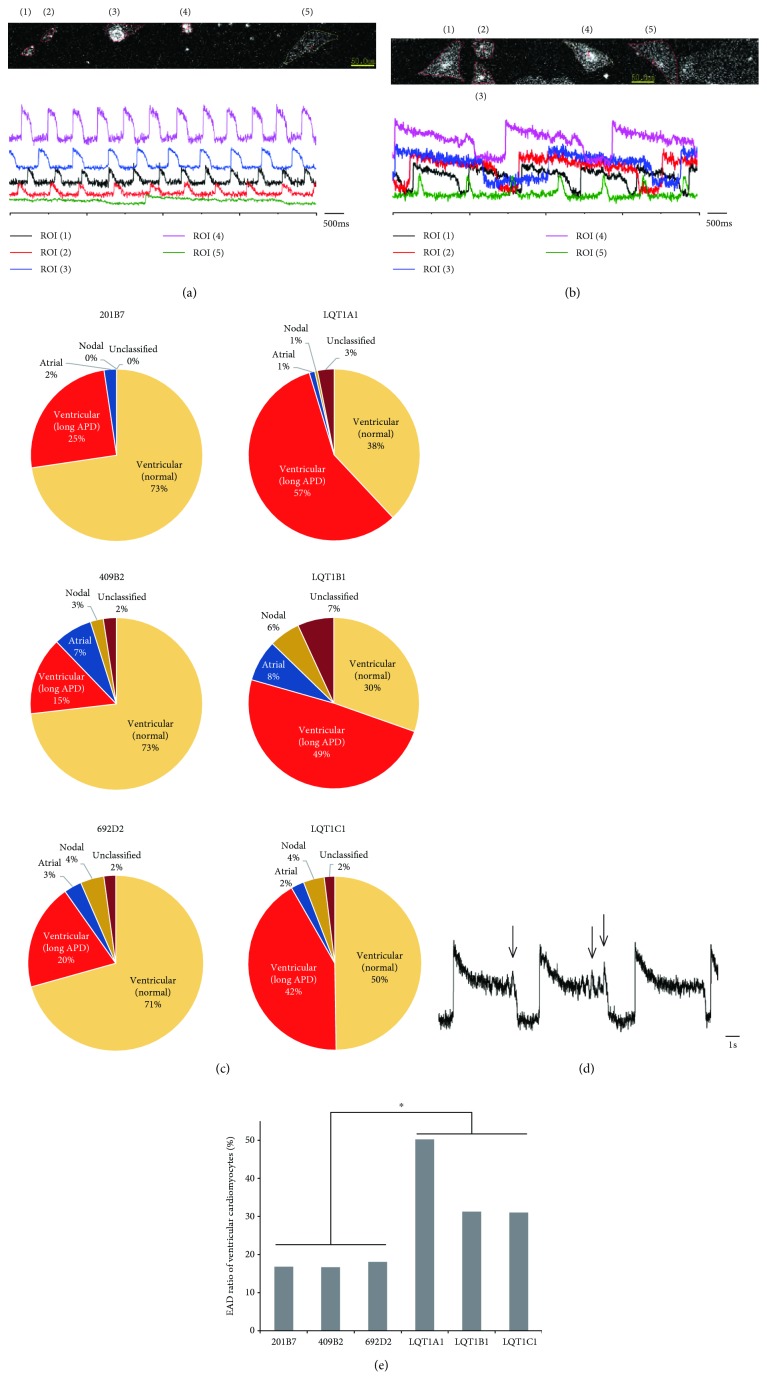
AP measurements of single cells using a membrane potential dye. (a) Examples of AP waves from control (409B2) measured by the dye. ROIs (1)–(5) are ventricular type. ROI (5) (green wave) shows long APD. (b) Examples of AP waves from LQT1B1. ROIs (1)–(4) are ventricular type with long APD. ROI (4) (purple wave) shows EADs. ROI (5) (green wave) is nodal type. (c) Proportion of subtypes in 3 control- and 3 LQT1-iPSC-CMs: 201B7 (*n* = 128), 409B2 (*n* = 41), 692D2 (*n* = 92), LQT1A1 (*n* = 192), LQT1B1 (*n* = 101), and LQT1C1 (*n* = 202). Ventricular-type cells predominate in all six lines. (d) Representative EADs in a ventricular-type cell from LQT1B1. The EADs were defined as over 20% of the AP amplitude. Arrows show EADs. (e) Occurrence ratio of EADs in ventricular cardiomyocytes from the six lines. The number of ventricular cells was used as the denominator; ^∗^ *p* < 0.05.

We classified the cardiomyocytes into subtypes based on the APs of single cells obtained by FV (Figures 3(a) and 3(b) and Supplementary Figure 2). Ventricular, atrial, and nodal cardiomyocytes were defined as APD_90_/APD_50_ < 1.4, 1.7 < APD_90_/APD_50_, and 1.4 < APD_90_/APD_50_ < 1.7, respectively, as previously reported (Supplementary Figure 2) [27, 28]. We labeled ventricular cells whose APD was more than 1 second as “ventricular cells with long APD.” These cells were more frequently observed among LQT-iPSC-CMs (Figures 3(b) and 3(c)) than control-iPSC-CMs (Figures 3(a) and 3(c)). The ventricular-like cardiomyocytes in control and LQT1-iPSC-CMs constituted the major population (201B7 (control), 98%; 409B2 (control), 88%; 692D2 (control), 90%; LQT1A1 (mother), 95%; LQT1B1 (elder sister), 79%; and LQT1C1 (younger sister), 93%) (Figure 3(c)). The frequencies of ventricular cells with long APD in control-iPSC-CMs (201B7, 16%; 409B2, 17%; and 692D2, 19%) were lower than those in LQT1-iPSC-CMs (LQT1A1, 50%; LQT1B1, 31%; and LQT1C1, 32%) (Figure 3(c)). Intriguingly, we observed more early afterdepolarizations (EADs) in LQT1-iPSC-CM populations than in control-iPSC-CM populations (Figures 3(d) and 3(e)) (*p* value, 0.031).

### 3.4. Action Potentials Recorded by FV in hiPSC-CM Monolayers

**Figure 4:**
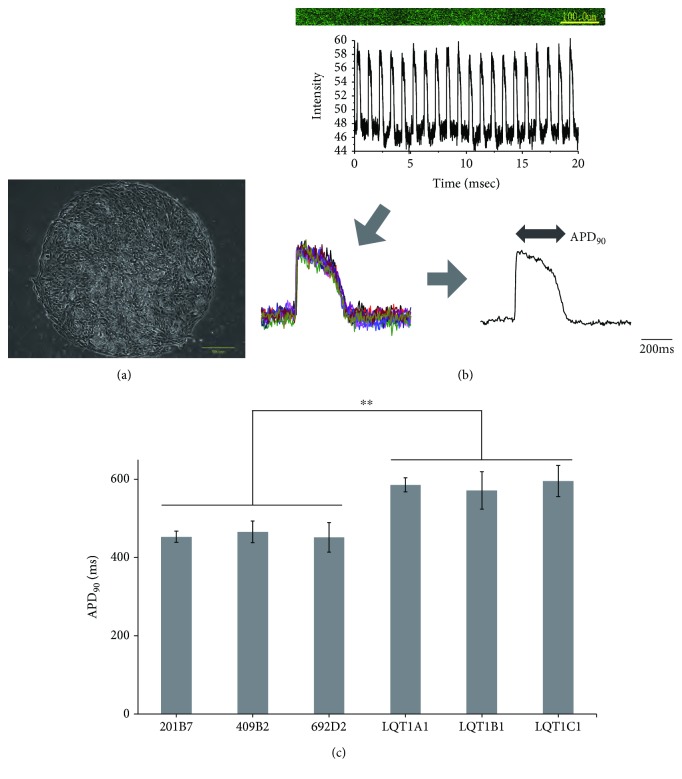
AP measurements in a cardiomyocyte monolayer. Data are represented as means ± SEM. (a) An example phase contrast image of the monolayer culture. Scale bar, 500 *μ*m. (b) Representative AP of a paced control-iPSC-CM monolayer. APD_90_ was calculated from the average of 10 consecutive waves. (c) APD_90_ from 3 control- and 3 LQT-iPSC-CM monolayers: 201B7 (*n* = 3), 409B2 (*n* = 3), 692D2 (*n* = 3), LQT1A1 (*n* = 3), LQT1B1 (*n* = 3), and LQT1C1 (*n* = 3). LQT-iPSC-CMs showed longer APD than controls; ^∗∗^ *p* < 0.005.

We next measured the APDs during pacing in high-density cardiomyocyte culture (Figure 4(a)). Ten consecutive waves after dye loading were averaged (Figure 4(b) and Supplementary Movie). APD_90_ from LQT1-iPSC-CMs was significantly longer than that from control-iPSC-CMs (Figure 4(c)) (*p* value, 0.000096).

### 3.5. APD Measured by FV on High-Throughput Plate Reader

**Figure 5:**
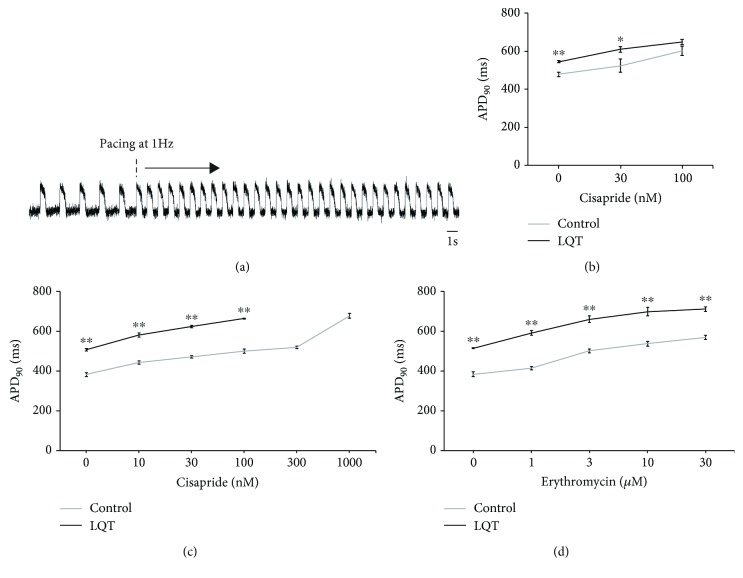
AP measurements in cardiomyocyte monolayers using a high-throughput plate reader. (a) Representative AP waves on a 96-well plate. Pacing at 1 Hz starts 10 s after recording. (b) Cisapride prolonged APD_90_ of both control- and LQT1-iPSC-CMs; 692D2 (control) (*n* = 3), LQT1B1 (*n* = 5). (c) Cisapride prolonged APD_90_ of both control- and LQT1-iPSC-CMs with 100 nM isoproterenol; 692D2 (*n* = 5), LQT1B1 (*n* = 3). (d) Erythromycin prolonged APD_90_ of both control- and LQT1-iPSC-CMs with 100 nM isoproterenol; 692D2 (*n* = 4), LQT1B1 (*n* = 3). LQT-iPSC-CM monolayers in 300 nM and 1000 nM cisapride were not synchronized at 1 Hz pacing. Data are represented as means ± SEM; ^∗∗^ *p* < 0.005, ^∗^ *p* < 0.05.

We next measured hiPSC-CM monolayers by FV in a higher throughput system consisting of a 96-well plate. The APs on monolayers were stable before and after 1 Hz pacing (Figure 5(a)). The APD_90_ averaged from 10 consecutive waves of LQT1B1-iPSC-CMs was longer than that from control-iPSC-CMs, and cisapride prolonged the APD_90_ of cardiomyocytes derived from both iPSC groups (Figure 5(b)). In addition, we administered a beta stimulant to mimic the conditions under exertional or emotional stress, in which the disease phenotypes are more prominent [5, 6]. In the presence of 100 nM isoproterenol (ISP), we assessed the effect of several agents on drug-induced long QT syndrome (diLQTS). Erythromycin and cisapride prolonged APD_90_ in both iPSC-CM groups under ISP, but the APD_90_ of LQT-iPSC-CMs was consistently higher at all concentrations (Figures 5(c) and 5(d)). These findings support the applicability of the membrane dye system to high-throughput-based drug discovery and toxicology testing.

## 4. Discussion

The proportion of a cardiac subtype in iPSC-CMs or cardiac monolayers and the AP parameters were reported to vary depending on the culture duration [29], suggesting the importance of a high proportion of the ventricular subtype when modeling the disease phenotypes of ventricular arrhythmias. In this study, we have proposed a simple method that uses FV to measure both single cardiomyocytes and cardiac monolayers derived from normal and LQT1-iPSCs. Optical recordings of single cardiomyocytes identified the cardiac subtypes of each cells and confirmed their variable electrophysiological properties (Figures 3(a)–3(c)), which was consistent with previous reports of a genetically encoded membrane potential sensor [15, 16]. High electrophysiological variability of single iPSC-CMs warrants assessment of a large population of ventricular cells for modeling ventricular arrhythmia. Our results demonstrated the feasibility of optical recording to identify cardiac subtypes and assess the electrophysiological properties of a large number of iPSC-CMs simultaneously.

The monolayered cells successfully reduced the variations of APD when synchronized with electrical stimulation at a constant frequency. As previously reported, FPD, an electrophysiological parameter highly correlated with APD [30], of ventricular hiPSC-CM monolayers was longer than that of atrial iPSC-CM monolayers [9], suggesting that the high proportion of the ventricular subtype would be appropriate for analyzing the APs of LQT-iPSC-CM monolayers and diLQTS models. Combined with single cell-based identification of cardiac subtypes, our optical recordings confirmed that iPSC-CM monolayers composed mainly of ventricular cells recapitulated the prolonged AP duration of cLQTS and diLQT. In addition, this system was applicable to high-throughput analysis to investigate the response to drugs.

Although conventional styryl voltage-sensitive dyes, such as di-4-ANEPPS and di-8-ANEPPS, have the ability to respond quickly to voltage changes, they have low sensitivity and nonnegligible phototoxicity [17, 31]. FV and di-4-ANBDQBS, a near-infrared fluorescent voltage-sensitive dye, can precisely track the transmembrane voltage with lower photodynamic damage than di-4-ANEPPS [12, 32, 33]. Furthermore, FV has been used for a high-throughput screening [19].

ArcLight is a genetically encoded sensor of membrane potential that has brighter fluorescence and negligible phototoxicity and has been used to study cardiomyocytes derived from human embryonic stem cells [15] and LQT2 iPSC-CMs [16]. However, the recorded APs do not agree with those from patch clamp techniques due to the slow temporal response of ArcLight [34]. VSFP-CR [35], a derivative of VSFP2.3 [36], was previously combined with subtype-specific marker genes (*MLC2v*, *SLN*, and *SHOX2*) to analyze the APs of three iPSC-CM subtypes [37]. Although genetically encoded voltage indicators enabled promotor-specific voltage response, it takes much time to establish the genetically modified cell lines stably. On the other hand, FV can be rapidly used with many cell lines, as it needs only 20 min loading.

diLQTS, which is an acquired LQTS, is mainly caused by blockage of the I_Kr_ channel and is more frequent than cLQTS. It is well known that the mutation of genes responsible for cLQTS could contribute to an increased risk of diLQTS [38, 39]. diLQTS has a similar mutation rate as cLQTS for the three major genes responsible for cLQTS [40], leading to the hypothesis that type 1 cLQTS patient-derived iPSC-CMs are appropriate for studying drugs that cause QT prolongation by blocking I_Kr_ current. In the present study, we used FV to identify drugs that prolonged QT in LQT1-iPSC-CM and control-iPSC-CM monolayers in a high-throughput manner (Figures 5(b)–5(d)).

Despite our encouraging results, it should be noted that FV has some limitations because the measured APs are based on a relative scale. Nor do the APs provide MDP or APA values, unlike patch clamp analysis. Furthermore, hiPSC-CMs are heterogeneous in electrophysiological properties, including their APDs and automaticities, making it difficult to measure the APs of paced cardiomyocytes simultaneously. Thus, in FV experiments, APDs in single cells were obtained from unpaced cells with different beating frequencies. Heterogeneity of the induced cardiomyocytes may also cause variations in the recordings. Studies have investigated ways to mature hiPSC-CMs [41, 42], which could reduce the heterogeneity and thus contribute to more robust and precise high-throughput screening.

Although many reports have used the AP morphology-based classification of iPSC-CM subtypes [27, 28, 43, 44], it has recently been argued that AP morphology is not a reliable indicator [45, 46]. Future methods that can distinguish cardiomyocyte subtypes induced from pluripotent stem cells would contribute to measuring long APD in LQT-iPSC-CMs more precisely.

## 5. Conclusions

In summary, using an FV-based optical measurement system, we successfully identified ventricular-type cells as the major population in cardiac subtypes and found that they have a higher frequency of APD prolongation and EAD when derived from LQT1 patient iPSCs. Further, monolayers with this major population were suitable for analyzing two-dimensional AP in LQT-iPSC-CMs and diLQTS models in a high-throughput manner.

## Data Availability

The data used to support the findings of this study are available from the corresponding author upon request.

## Supplementary Materials

Supplementary 1 Supplementary Figure 1: the expression of genes related to action potential duration in control- and LQT1-iPSC-cardiomyocytes. Supplementary Figure 2: action potentials of ventricular-, atrial-, and nodal-type cardiomyocytes recorded by FluoVolt dye.

Supplementary 2 Supplementary Movie: a blinking cardiomyocyte monolayer treated by FluoVolt dye.
