# Erratum to “Optical Recording of Action Potentials in Human Induced Pluripotent Stem Cell-Derived Cardiac Single Cells and Monolayers Generated from Long QT Syndrome Type 1 Patients”

**DOI:** 10.1155/2020/8765895

**Published:** 2020-01-23

**Authors:** Tadashi Takaki, Azusa Inagaki, Kazuhisa Chonabayashi, Keiji Inoue, Kenji Miki, Seiko Ohno, Takeru Makiyama, Minoru Horie, Yoshinori Yoshida

**Affiliations:** ^1^Department of Cell Growth and Differentiation, Center for iPS Cell Research and Application, Kyoto University, Sakyo-ku, Kyoto 606-8507, Japan; ^2^Department of Cardiology, Japanese Red Cross Kyoto Daini Hospital, Kamigyo-ku, Kyoto 602-8026, Japan; ^3^Department of Bioscience and Genetics, National Cerebral and Cardiovascular Center Research Institute, Suita, Osaka 565-8565, Japan; ^4^Department of Cardiovascular Medicine, Kyoto University Graduate School of Medicine, Sakyo-ku, Kyoto 606-8501, Japan; ^5^Center for Epidemiologic Research in Asia, Shiga University of Medical Science, Seta-Tsukinowa-cho, Otsu 520-2192, Japan

In the article titled “Optical Recording of Action Potentials in Human Induced Pluripotent Stem Cell-Derived Cardiac Single Cells and Monolayers Generated from Long QT Syndrome Type 1 Patients” [1], there were errors in Figure 1(a) where extra lines were drawn during the production process. The corrected figure is shown below:

## Figures and Tables

**Figure 1:**
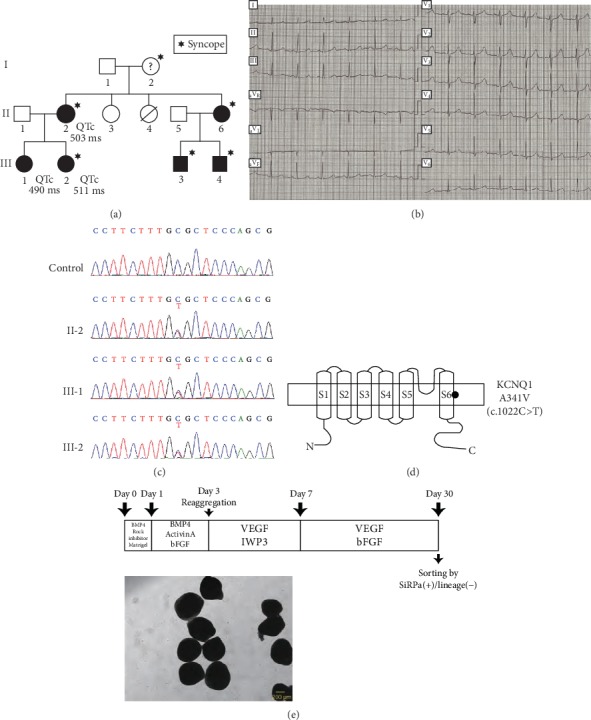
Type 1 long QT syndrome family background and cardiac differentiation from human iPSCs. (a) Family pedigree. The squares indicate males, and the circles indicate females. Closed symbols mark patients confirmed by their DNA sequences. Hexagrams mark members who have a syncope history. The QTc values of three patients before taking a beta-blocker are stated. (b) ECG of II-2 in (a) before the patient started taking a beta-blocker. (c) Sanger sequencing of the three patients and one control. (d) Schematic figure of KCNQ1 protein. The black circle indicates the mutation site within the transmembrane region. The lower side locates intracellular. (e) Outline of the cardiac differentiation. Lower: representative shapes of beating EBs. Scale bar: 200 *μ*m.
