# Supplementary material for: Optical Recording of Action Potentials in Human Induced Pluripotent Stem Cell-Derived Cardiac Single Cells and Monolayers Generated from Long QT Syndrome Type 1 Patients
Source: Stem Cells Int. 2019 Mar 6;2019:7532657. doi: 10.1155/2019/7532657 (PMC6431403; doi:10.1155/2019/7532657)
Supplement: Supplementary 1 — Supplementary Figure 1: the expression of genes related to action potential duration in control- and LQT1-iPSC-cardiomyocytes. Supplementary Figure 2: action potentials of ventricular-, atrial-, and nodal-type cardiomyocytes recorded by FluoVolt dye. [file 7532657.f1.docx]

**Supplementary Methods**

In vitro cardiac differentiation

Human iPSCs were differentiated by forming EBs [1, 2]. On day 0, undifferentiated iPSCs were dissociated with Accumax (Innovative Cell Technologies, San Diego, CA, USA), and 5,000 cells were aggregated using a 96-well plate coated with Ultra-Low Attachment Surface, round bottom (Corning, Corning, NY, USA) in StemPro34 SFM (Thermo Fisher Scientific, Waltham, MA, USA) supplemented with buffer (2 mM L-glutamine (Thermo Fisher Scientific), 4x10-4 M monothioglycerol (MTG) (Sigma-Aldrich, St Louis, MO, USA), 50 μg/ml ascorbic acid (Sigma-Aldrich), 150 μg/ml transferrin (Roche Diagnostics, Basel, Swiss), 50 U/ml penicillin, and 50 μg/ml streptomycin), 10 μM Y-27632 (WAKO, Osaka, Japan), 2 ng/ml BMP4 (R&D Systems, Minneapolis, MN, USA), and 0.5% BD Matrigel Matrix Growth Factor Reduced (BD Biosciences, San Jose, CA, USA). On day 1, medium, including human recombinant activin A (12 ng/ml) (R&D Systems), BMP4 (18 ng/ml), and bFGF (10 ng/ml) (R&D Systems), were added into the wells to reach a final concentration of 6 ng/ml activin A, 10 ng/ml BMP4, and 5 ng/ml bFGF. On day 3, EBs were collected, dissociated with Accumax, and suspended in differentiation medium supplemented with buffer plus 10 ng/ml VEGF (R&D Systems) and 1 ng/ml IWP-3 (Stemgent, Lexington, KY, USA). 10,000 cells were aggregated in a new 96-well plate coated with Ultra-Low Attachment Surface, round bottom. On day 7, EBs were picked up into a 6-well or 10-cm plate coated with Ultra-Low Attachment Surface, and the medium was changed to StemPro 34 SFM containing buffer plus 10 ng/ml VEGF and 5 ng/ml bFGF. For the maintenance of iPSC-CMs, the culture medium was renewed every 2-3 days. Day 0-12 EBs were incubated at 37°C, 5% O_2_, 5% CO_2_, and 90% N_2_. From day 12, the condition was changed to 37°C, 5% CO_2_, and 95% air.

Quantitative polymerase chain reaction (qPCR)

Total RNA was extracted and purified from cells after FACS sorting using QIAzol Lysis Reagent (QIAGEN, Hilden, Germany) with the miRNeasy Mini Kit (QIAGEN) according to the manufacturer’s protocol. The RNA concentrations were determined using NanoDrop 2000 (Thermo Fisher Scientific). Reverse transcription reaction from purified RNA was performed using ReverTra Ace (Toyobo, Osaka, Japan) with the oligo dT20 primer. qRT-PCR was performed with TaqMan gene expression assays (Thermo Fisher Scientific) using the StepOnePlus Real-Time PCR system (Thermo Fisher Scientific). Expression levels were normalized against the expression level of an internal housekeeping gene (*ACTB*). The qPCR Taqman probes used in this study were as follows.

*CACNA1C*: Hs00167681_m1, *RYR2*: Hs00181461_m1, *SLC8A1*: Hs01062258_m1, *KCNQ1*: Hs00923522_m1, *KCNE1*: Hs00897540_s1, *KCNH2*: Hs04234270_g1, *KCNE2*: Hs00270822_s1, *SCN5A*: Hs00165693_m1, and *ACTB*: Hs99999903_m1.

Current clamp recording

Perforated whole-cell patch was performed with current clamp mode using an Axopatch 200B amplifier (Molecular Devices, Sunnyvale, CA, USA) and pCLAMP software (v9.2 or 10.4; Molecular Devices) with the IX73 inverted microscope (Olympus, Tokyo, Japan) and phase contrast objectives UPLFLN10XPH and UPLFLN20XPH (Olympus). Patch electrodes were made of borosilicate capillaries (1B150F-4; WPI, Sarasota, FL, USA) using a micropipette puller (P-97/IVF or P-1000IVF, Sutter Instruments, Novato, CA, USA). The level of stretching and heating was adjusted to obtain a resistance of 2–4 MΩ. The patch electrodes were filled with 300-500 μg/mL amphotericin B (Sigma-Aldrich) plus intracellular solution containing 130 mM KOH, 130 mM L-Aspartic acid, 20 mM KCl, 5 mM NaCl, 10 mM HEPES, 5 mM Mg-ATP, 10 mM EGTA, and 1 mM MgCl_2_. The pH was adjusted to 7.2 with KOH. APs were recorded from iPSC-CMs superfused with modified Tyrode’s solution, which consisted of 120 mM NaCl, 5 mM KCl, 2 mM CaCl_2_, 1 mM MgCl_2_, 0.84 mM Na_2_HPO_4_, 0.28 mM MgSO_4_, 0.22 mM KH_2_PO_4_, 27 mM NaHCO_3_, and 5.5 mM glucose, pH7.4, at 35-37°C. After the Axopatch amplifier was set to current clamp mode, spontaneous APs were recorded from spontaneously contracting hiPSC-CMs 5-10 days after seeding using the gap-free protocol, and paced APs were recorded from spontaneously contracting or quiescent cells using a pacing protocol of 4 ms duration, 0.2-1 nA amplitude, and 1 Hz frequency. All signals were filtered at 5 kHz, digitized at 10 kHz using Digidata 1322A or 1550A (Molecular Devices), and analyzed with pCLAMP 9.2 or 10.4 software (Molecular Devices). MDP, APA, and APD_90_ were calculated from the average AP of 10 consecutive and stable waves in pacing mode.

Voltage clamp recording

I_Ks_ currents were recorded from single cardiomyocytes 5-10 days after seeding with the ruptured whole cell patch clamp method [3]. I_Ks_ currents were calculated by subtracting the currents after perfusion with extracellular solution containing 30 µM (-)-[3R,4S]-chromanol 293B (Tocris Bioscience, Bristol, UK) from the currents before perfusion. The extracellular solution consisted of 150 mM NaCl, 5.4 mM KCl, 1.8 mM CaCl_2_, 1 mM MgCl_2_, 1 mM Na-pyruvate, 15 mM HEPES, and 1 µM nifedipine, pH7.4. The extracellular solution was perfused at 35-37°C using a peristaltic pump, MINIPULS 3, type MP-2 (Gilson, Middleton, WI, USA). The patch electrode was filled with the internal pipette solution containing 125 mM K-Aspartate, 20 mM KCl, 10 mM EGTA, 5 mM MgATP, 5 mM HEPES, 1 mM MgCl_2_, 2 mM Na_2_-phosphocreatinine, and 2 mM Na_2_-GTP, pH7.2. Patch electrodes were made of borosilicate capillaries (1B150F-4) using a micropipette puller (P-97/IVF) whose level of stretching and heating was adjusted to obtain a resistance of 4-6 MΩ.

Data were acquired at 10 kHz with Digidata 1322A, low-pass filtered at 2 kHz with Axopatch 200B amplifier, and filtered at 1 kHz with Clampfit software (Molecular Devices) after recording. I_Ks_ currents were elicited by depolarizing steps from a holding potential of -40 mV to −20, 0, 20, and 40 mV for 4 s. This was followed by a 2-s repolarization phase.

Optical recording of APs from single cells or cardiomyocyte monolayers

Cells were imaged in a 35-mm-diameter glass bottom dish at 37°C and modified Tyrode’s solution identical to that used for patch clamp recordings fed with 5% CO_2_, 20% O_2_, and 75% N_2_ perfused through water at 37°C using Humidification Bottle (Carl Zeiss, Jena, Germany) and Heating Device Humidity S1 (Carl Zeiss). One hour after setting the cells, fluorescence measurements were taken with the Axio Observer inverted fluorescence microscope (Carl Zeiss), Objective Fluar 10x/0.50 (Carl Zeiss), Xe lamp C7773 (Hamamatsu Photonics), EM-CCD camera ImagEM (Hamamatsu Photonics), filter set zeiss 16 (Excitation: BP 485/20, Beam Splitter: FT510), emission filter 535DF55 (Omega optical, Brattleboro, VT, USA), and AquaCosmos2.6 software (Hamamatsu Photonics) set at Ex 490 BP 10 nm. The Xe lamp was set at 100% output. Subarray images were recorded every 8 ms in single cells 5-10 days after seeding or every 4 ms in monolayer 10-15 days after seeding. Binning was set to 1x1. The regions of interest (ROIs) in the monolayers were defined as whole pixels of 512x32. The monolayers were stimulated at 1 Hz with 1 ms depolarizing pulses at 10 V using a pulse stimulator, Master-9 (A.M.P.I., Jerusalem, Israel), with an interelectrode distance of 12 mm (Intermedical, Osaka, Japan) [4]. Graphing, superimposed waveform traces, and calculation of APD_90_ were performed with OriginPro 2016 (OriginLab, Northampton, MA, USA).

**Supplementary Figures**

**Supplementary Figure 1.**

qPCR analysis after FACS sorting of SIRPa(+)/lineage(-) at day 30 from 692D2 (n=3) and LQT1A1 (n=3). The relative gene expression was set to 1 for genes that affect ion currents in control line 692D2. There was no significant difference in the expression of any genes between 692D2- and LQT1A1-hiPSC-CMs. Data are represented as means ± SEM. Expression levels were normalized against the expression level of an internal housekeeping gene (*ACTB*).

**Supplementary Figure 2.**

Action potentials of three major cardiomyocyte subtypes from LQT1B1-iPSCs recorded by FluoVolt dye. ROI (1), (2), and (3) show ventricular-, atrial-, and nodal-type cardiomyocytes, respectively.

**Supplementary Reference**

[1]. S. Funakoshi, K. Miki, T. Takaki et al., "Enhanced engraftment, proliferation, and therapeutic potential in heart using optimized human ipsc-derived cardiomyocytes," *Sci Rep*, vol. 6, pp. 19111, 2016.

[2]. K. Miki, K. Endo, S. Takahashi et al., "Efficient Detection and Purification of Cell Populations Using Synthetic MicroRNA Switches," *Cell Stem Cell*, vol. 16, no. 6, pp. 699-711, 2015.

[3]. J. Ma, L. Guo, S. J. Fiene et al., "High purity human-induced pluripotent stem cell-derived cardiomyocytes: electrophysiological properties of action potentials and ionic currents," *Am J Physiol Heart Circ Physiol*, vol. 301, no. 5, pp. H2006-2017, 2011.

[4]. K. Sasaki, T. Makiyama, Y. Yoshida et al., " Patient-Specific Human Induced Pluripotent Stem Cell Model Assessed with Electrical Pacing Validates S107 as a Potential Therapeutic Agent for Catecholaminergic Polymorphic Ventricular Tachycardia ," *PLoS One*, vol. 11, no. 10, pp. e0164795, 2016.
